# Porcine Reproductive and Respiratory Syndrome Virus Utilizes Viral Apoptotic Mimicry as an Alternative Pathway To Infect Host Cells

**DOI:** 10.1128/JVI.00709-20

**Published:** 2020-08-17

**Authors:** Xin Wei, Rui Li, Songlin Qiao, Xin-xin Chen, Guangxu Xing, Gaiping Zhang

**Affiliations:** a College of Animal Science and Veterinary Medicine, Henan Agricultural University, Zhengzhou, Henan, China; b Key Laboratory of Animal Immunology of the Ministry of Agriculture, Henan Provincial Key Laboratory of Animal Immunology, Henan Academy of Agricultural Sciences, Zhengzhou, Henan, China; University of Texas Southwestern Medical Center

**Keywords:** PRRSV, TIM, macropinocytosis, phosphatidylserine, viral apoptotic mimicry

## Abstract

Porcine reproductive and respiratory syndrome (PRRS), caused by PRRS virus (PRRSV), has led to enormous economic losses in global swine industry. Infection by PRRSV is previously shown to be via low pH-dependent clathrin-mediated endocytosis, and CD163 functions as an essential receptor during viral infection. Despite much research focusing on it, PRRSV infection remains to be fully elucidated. In this study, we demonstrated that PRRSV externalized phosphatidylserine (PS) on the envelope as viral apoptotic mimicry and infected host cells through T-cell immunoglobulin and mucin domain (TIM)-induced and CD163-involved macropinocytosis as an alternative pathway. In detail, we identified that PS receptor TIM-1/4 recognized and interacted with PRRSV as viral apoptotic mimicry and subsequently induced macropinocytosis by the downstream Rho GTPases Rac1, cell division control protein 42 (Cdc42), and p21-activated kinase 1 (Pak1). Altogether, these results expand our knowledge of PRRSV infection, which will support implications for the prevention and control of PRRS.

**IMPORTANCE** PRRS has caused huge economic losses to pig farming worldwide. Its causative agent, PRRSV, infects host cells through low pH-dependent clathrin-mediated endocytosis and CD163 is indispensable during the process. Whether there exist alternative infection pathways for PRRSV arouses our interest. Here, we found that PRRSV exposed PS on its envelope and disguised as apoptotic debris. The PS receptor TIM-1/4 recognized PRRSV and induced the downstream signaling pathway to mediate viral infection via CD163-dependent macropinocytosis. The current work deepens our understanding of PRRSV infection and provides clues for the development of drugs and vaccines against the virus.

## Abstract (precis)

PRRS has caused huge economic losses to pig farming worldwide. Its causative agent, PRRSV, infects host cells through low pH-dependent clathrin-mediated endocytosis and CD163 is indispensable during the process. Whether there exist alternative infection pathways for PRRSV arouses our interest. Here, we found that PRRSV exposed PS on its envelope and disguised as apoptotic debris. The PS receptor TIM-1/4 recognized PRRSV and induced the downstream signaling pathway to mediate viral infection via CD163-dependent macropinocytosis. The current work deepens our understanding of PRRSV infection and provides clues for the development of drugs and vaccines against the virus.

## INTRODUCTION

As intracellular obligate pathogens, both DNA and RNA viruses have evolved diverse strategies to infect host cells for productive replication (1, 2). A variety of viruses incorporate phosphatidylserine (PS), a marker of apoptosis (3), on the surfaces of their envelopes and disguise as apoptotic debris. Upon recognition by PS receptors (PSRs) and induction of downstream signaling cascades, these viruses are internalized via clathrin-mediated endocytosis (CME) and/or macropinocytosis by host cells to promote their infections (4, 5), namely, viral apoptotic mimicry (6).

For the viruses utilizing apoptotic mimicry, diverse PSRs have been identified, including T-cell immunoglobulin and mucin domain 1/3/4 (TIM-1/3/4), brain-specific angiogenesis inhibitor 1 (BAI1), Stabilin-1/2, CD300a, TAM receptors (Tyro3, Axl or Mer) and integrins (αvβ3 or αvβ5) (7). For invasion routes, CME is constitutively driven by formation of clathrin-coated vesicles (8), while macropinocytosis is induced by extracellular stimuli and shows several characteristics, such as cytoskeletal rearrangement, fluid uptake, and dependence on Na^+^/H^+^ exchanger activity and Rho GTPases (9, 10).

Porcine reproductive and respiratory syndrome (PRRS) has become an economically critical factor in global swine industry since it was first reported in the United States in 1987 (11, 12). Currently, loss due to PRRS in the United States is annually estimated $664 million (13). Caused by PRRS virus (PRRSV), the syndrome is characterized by reproductive failures in the late-term gestation of sows and respiratory diseases in pigs of all ages (14). PRRSV is an enveloped single-stranded positive-sense RNA virus with a genome of approximately 15 kb (15, 16). All PRRSV isolates are classified into two genotypes, PRRSV-1 and PRRSV-2 (17), which belong to the order *Nidovirales*, family *Arteriviridae*, and genus *Porartevirus* (18).

PRRSV specially infects swine and the differentiated monocytes, particularly porcine alveolar macrophages (PAMs), are its primary target cells *in vivo* (19). In addition, the African green monkey kidney epithelial cell line MA-104 and its derivative, MARC-145, are susceptible to viral infection *in vitro* (20). Previous studies have shown that PRRSV infects host cells via low pH-dependent CME (21–23) and a scavenger receptor CD163 is indispensable for viral infection (24–27).

In the present work, we determined an alternative pathway utilized by PRRSV to infect host cells. First, we found that PRRSV exposed PS on the envelope as viral apoptotic mimicry. Next, we dissected the host cell PSRs recognizing PRRSV as apoptotic mimicry and explored the detailed mechanisms, including the downstream signaling pathways and invasion routes.

## RESULTS

### PRRSV externalizes PS on the envelope as viral apoptotic mimicry.

**FIG 1:**
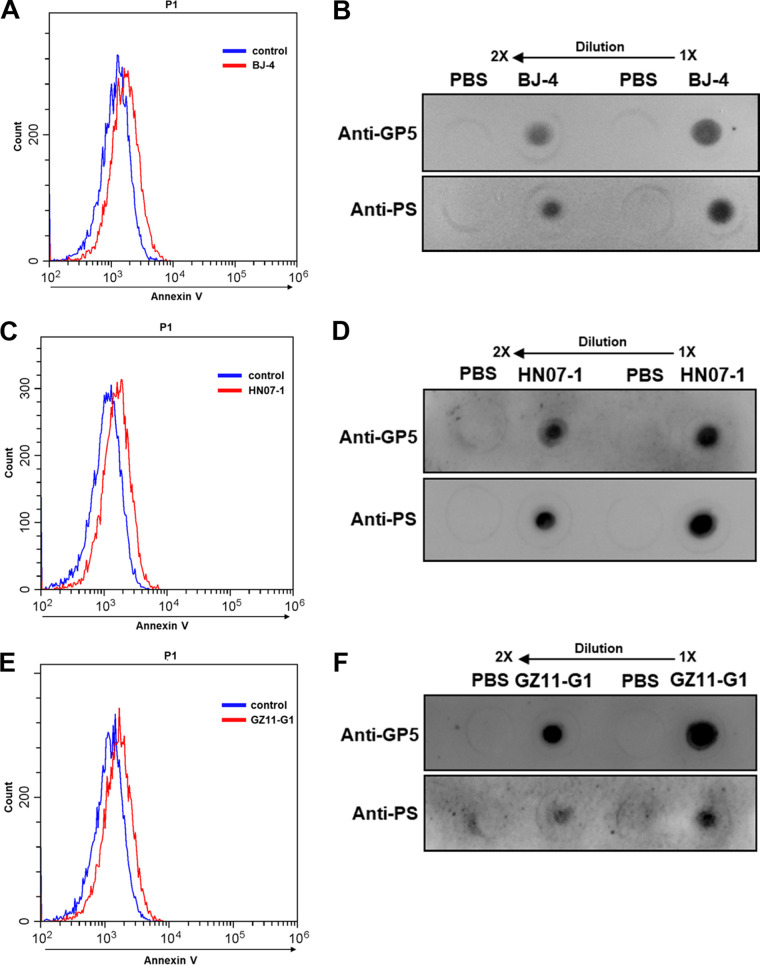
PRRSV externalizes PS on the envelope. PRRSV-2 strain BJ-4, HP-PRRSV strain HN07-1, and PRRSV-1 strain GZ11-G1 were shown to expose PS by FCM (A, C, and E) and dot blotting (B, D, and F). MARC-145 cells were inoculated with PRRSV (MOI = 10) at 4°C or PBS as an unbound control. Then, PS was assessed to PRRSV-bound or unbound cells by FCM immediately using annexin V-conjugated Alexa Fluor 488. Each experiment was independently performed three times with similar results, and data from one representative experiment are shown in panels A, C, and E. Dot blot assays were set up with anti-PS 1H6 MAb or anti-PRRSV GP5 MAb as the primary antibody. Twofold dilutions of purified PRRSV in PBS were applied for PS and PRRSV GP5 detection, respectively. PBS was spotted onto samples as a negative control.

In order to validate whether PRRSV incorporates PS on its envelope and utilizes viral apoptotic mimicry, we first detected PS on the virions using annexin V, a specific PS-binding protein (28), by flow cytometry (FCM). As shown in Fig. 1A, a typical PRRSV-2 strain, BJ-4, was externalized PS on the envelope. Furthermore, we exploited a commercial antibody against PS (29), a specific antibody against PRRSV major envelope glycoprotein (GP) 5 (30), and dot blotting to confirm that PRRSV BJ-4 did expose PS (Fig. 1B). In addition, highly pathogenic PRRSV (HP-PRRSV) strain HN07-1 and PRRSV-1 strain GZ11-G1 also externalized PS on the envelopes (Fig. 1C to F). All of these results demonstrate that PRRSV incorporates PS on the envelope surface as viral apoptotic mimicry. Since PRRSV-2 strains are predominantly prevalent and PRRSV-1 strains are sporadic in China (31), we only applied PRRSV BJ-4 to the following research.

### TIM-1 is identified to recognize PRRSV as apoptotic mimicry in MARC-145 cells.

**FIG 2:**
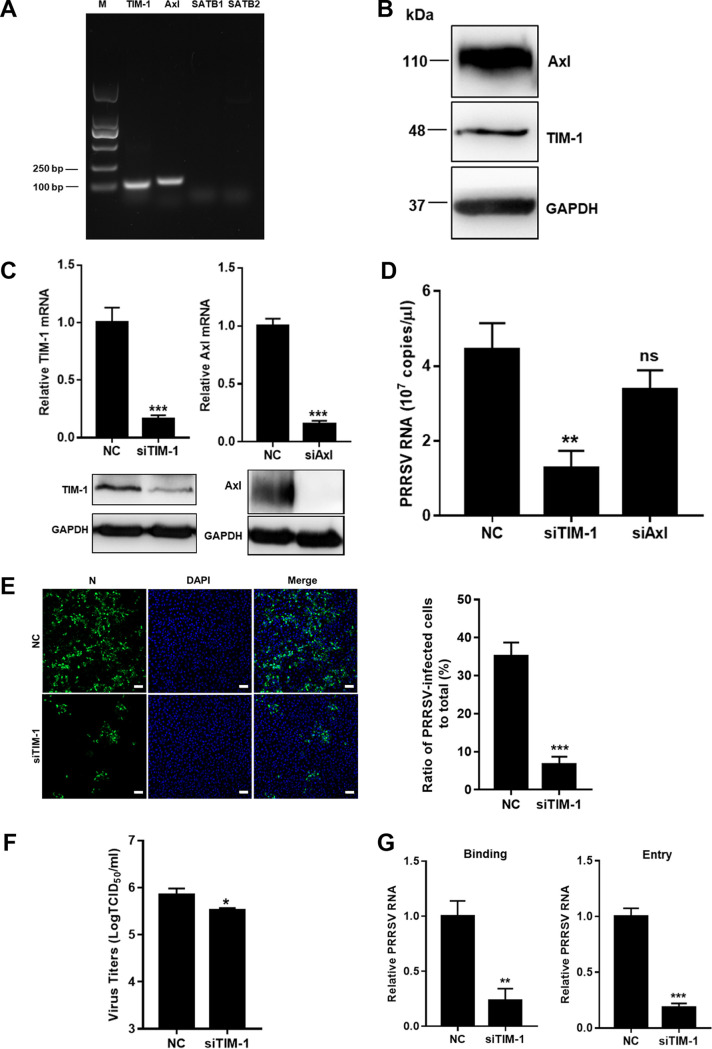
TIM-1 is identified to recognize PRRSV as apoptotic mimicry in MARC-145 cells. (A) Transcription of PSRs in MARC-145 cells. MARC-145 cells were collected, and the reverse transcription cDNAs were prepared and subjected to PCR with the specific primers of TIM-1, Axl, Stabilin-1, and Stabilin-2. The PCR products of each gene fragment were subjected to agarose gel electrophoresis. (B) Expression of TIM-1 and Axl as determined by IB analysis. MARC-145 cells were harvested and lysed. TIM-1 and Axl were detected by IB. Knockdown of TIM-1 (C) significantly influenced PRRSV RNA abundance (D), infectivity (E), progeny viral titers (F), and viral binding (G). MARC-145 cells were transfected with siTIM-1, siAxl, or siRNA-NC for 36 h and infected with PPRSV (MOI = 10). The infected cells were collected for analyses of PRRSV RNA by RT-qPCR at 12 hpi, N protein expression by immunofluorescence at 24 hpi, viral titers by determining the TCID_50_ at 48 hpi, or binding and entry by RT-qPCR. Immunofluorescence images were quantified by counting the number of cells expressing viral N protein. Four random fields were counted per each condition, and the total number of cells per field was determined by DAPI staining. Each experiment was performed three times, and similar results were obtained. Differences between groups were assessed by using a Student *t* test, and the statistical significance is indicated (*, *P* < 0.05; **, *P* < 0.01; ***, *P* < 0.001; ns, not significant). Scale bars, 50 μm.

PSRs TIM-1, Stabilin-1/2, and Axl are specially expressed in epithelial cells (7). Here, we sought to identify which host PSRs recognized PRRSV as apoptotic mimicry. Initially, we monitored the transcription of each PSR in MARC-145 cells. Figure 2A shows that TIM-1 and Axl were transcribed in the cells. Expression of TIM-1 and Axl were also demonstrated through immunoblotting (IB) analysis (Fig. 2B). To investigate their specific functions during PRRSV infection, knockdown of TIM-1 and Axl were carried out in MARC-145 cells (Fig. 2C). To measure total PRRSV RNA, a pair of internal primers in the viral open reading frame 7 (ORF7) gene were used to amplify all subgenomic mRNA and genomic RNA by quantitative real-time PCR (RT-qPCR) (32, 33). *Axl* knockdown did not significantly influence the abundance of PRRSV RNA (Fig. 2D). In contrast, PRRSV infection was suppressed, as indicated by decreased viral RNA abundance (3.5-fold) at 12 h postinfection (hpi), infectivity with nucleocapsid (N) protein expression (5-fold) at 24 hpi, and progeny viral titers (2.5-fold) at 48 hpi (Fig. 2D to F) in the *TIM-1* knockdown cells. Figure 2G further shows that knockdown of TIM-1 influenced PRRSV infection during viral binding to MARC-145 cells (4-fold).

**FIG 3:**
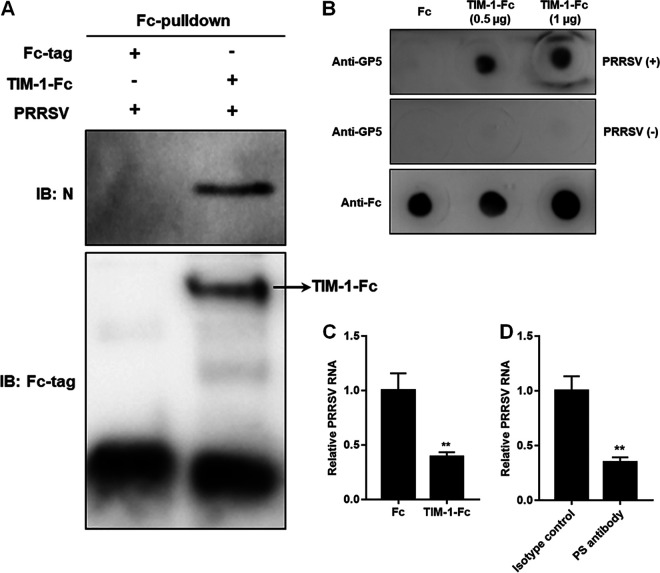
TIM-1 directly binds to PRRSV demonstrated by Fc-pulldown assay (A), dot blotting (B), viral binding interference (C), and a blocking experiment (D). For the Fc-pulldown assay, the recombinant TIM-1-Fc was bound to protein A/G-beads, whereas the Fc tag served as control. The beads were then incubated with the PRRSV virions. The eluted proteins were then subjected to IB. For dot blotting, TIM-1-Fc or Fc was spotted onto nitrocellulose membranes and detected by anti-human IgG antibody or anti-PRRSV GP5 MAb. After incubation with PRRSV BJ-4 for 4 h, the membranes were eaxmined for PRRSV GP5 detection. For viral binding interference, PRRSV BJ-4 virions were incubated with TIM-1-Fc (5 μg) or Fc at 4°C for 4 h, followed by inoculation into MARC-145 cells at 4°C for 1 h. For the blocking experiment, PRRSV BJ-4 virions were pretreated with anti-PS antibody or isotype control antibody at 4°C for 4 h and then inoculated into MARC-145 cells at 4°C for 1 h. The PRRSV RNA was determined by RT-qPCR. Each experiment was performed three times independently. Statistical analysis for the RT-qPCR was carried out using the Student *t* test (**, *P* < 0.01).

Next, we determined whether TIM-1 directly bound to PRRSV. *In vitro* Fc-pulldown assay with recombinant Fc-fused TIM-1 (TIM-1-Fc) and purified PRRSV indicated that TIM-1 bound to the virions (Fig. 3A). Dot blot analyses further confirmed that TIM-1 interacted with PRRSV (Fig. 3B). Incubation with recombinant TIM-1-Fc showed an interference of PRRSV binding to MARC-145 cells with decreased viral RNA (2.5-fold; Fig. 3C). A blocking experiment using the anti-PS antibody also showed a significant decrease in PRRSV binding to the cells (3-fold; Fig. 3D), suggesting the specific interaction.

Taken together, these data provide evidence that TIM-1 recognizes and interacts with PRRSV as apoptotic mimicry in MARC-145 cells.

### PRRSV induces macropinocytosis via TIM-1 in MARC-145 cells.

**FIG 4:**
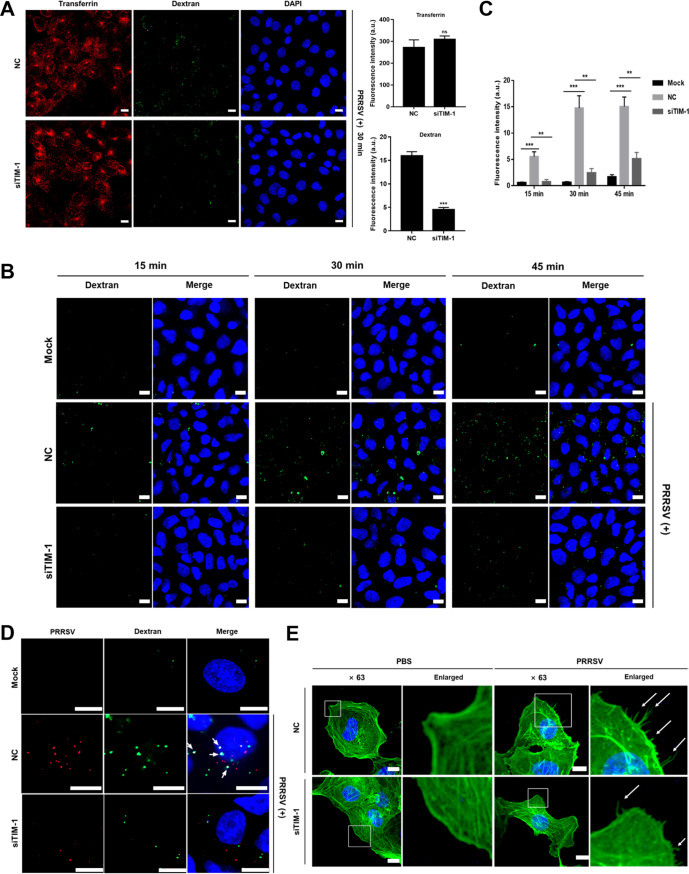
PRRSV induces macropinocytosis via TIM-1 in MARC-145 cells. (A) Knockdown of TIM-1 greatly influenced macropinocytosis. After transfection with siRNA-NC or siTIM-1 for 36 h, PRRSV BJ-4 (MOI = 10) was applied to the serum-starved MARC-145 cells at 4°C. The input virus was replaced with medium containing dextran (final concentration, 250 μg/ml) and transferrin (final concentration, 10 μg/ml) and transferred to 37°C for 30 min. The cells were then fixed, and the nuclei were stained with DAPI. Images were acquired with the same confocal microscope settings. The total fluorescence intensity of transferrin or dextran was calculated using ImageJ software. ***, *P* < 0.001; ns, not significant. (B) PRRSV induced macropinocytosis via TIM-1. MARC-145 cells were transfected with mock treatment, siRNA-NC, or siTIM-1 for 36 h and then serum starved for 2 h. The cells were inoculated with or without PRRSV BJ-4 (MOI = 10) at 4°C. The inoculum was replaced with medium containing FITC-dextran and transferred to 37°C for 15, 30, or 45 min. The cells were fixed and examined by confocal microscopy using the same confocal microscope settings. (C) The total fluorescence intensity of the dextran in panel B was calculated using ImageJ software. **, *P* < 0.01; ***, *P* < 0.001. (D) Serum-starved and siRNA-transfected MARC-145 cells were inoculated with PRRSV BJ-4 (MOI = 10) for 30 min, followed by dextran uptake (green). PRRSV infection is indicated by anti-PRRSV N protein antibody (red). White arrows indicate the colocalization of dextran and PRRSV. (E) PRRSV induced membrane protrusions. The serum-starved MARC-145 cells were added with PRRSV BJ-4 (MOI = 10) or PBS for 30 min and fixed with 4% PFA. Actin filaments were labeled with phalloidin (green). Images were captured with a 63× oil immersion objective. A higher magnification of the boxed area shows the formation of actin protrusions on the cell surface (white arrows). Scale bars, 10 μm.

As apoptotic mimicry, viruses infect host cells via CME and/or macropinocytosis (34, 35). To distinguish which routes were involved in PRRSV infection mediated by TIM-1, we performed confocal microscopy in combination with transferrin or dextran during early infection (i.e., at 30 min postinfection [mpi]) in MARC-145 cells. Transferrin is a marker for CME (36), and dextran is a fluid-phase marker which is robustly internalized during macropinocytosis (37). As shown in Fig. 4A, knockdown of TIM-1 greatly influenced macropinocytosis (3.5-fold) but not CME, suggesting that PRRSV induced macropinocytosis via TIM-1. To support this conclusion, we conducted the assay once again with dextran using confocal microscopy for different time periods. In Fig. 4B and C, PRRSV induced macropinocytosis at as early as 15 mpi, while *TIM-1* knockdown inhibited PRRSV-induced macropinocytosis (3- to 7-fold). We further demonstrated this conclusion using confocal microscopy simultaneously with the specific antibody against PRRSV N protein and dextran at 30 mpi, which also indicated their colocalization during the process (Fig. 4D).

Macropinocytosis is distinct from other endocytic pathways in extensive actin rearrangements and the formation of protrusions on cellular surfaces (9, 10). To determine whether PRRSV induces membrane protrusions, we performed confocal microscopy with phalloidin and monitored actin restructuring. Phalloidin specially binds to the polymerized form of actin (38). As shown in Fig. 4E, PRRSV infection led to depolymerization and distribution changes of actin. More actin-driven membrane protrusions were observed on cell surfaces of MARC-145 cells than that of mock-infected cells (Fig. 4E, white arrows). However, there were decreased membrane protrusions in *TIM-1* knockdown MARC-145 cells.

These results illustrate that PRRSV induces macropinocytosis via TIM-1 in MARC-145 cells.

### PRRSV utilizes macropinocytosis to infect MARC-145 cells.

**FIG 5:**
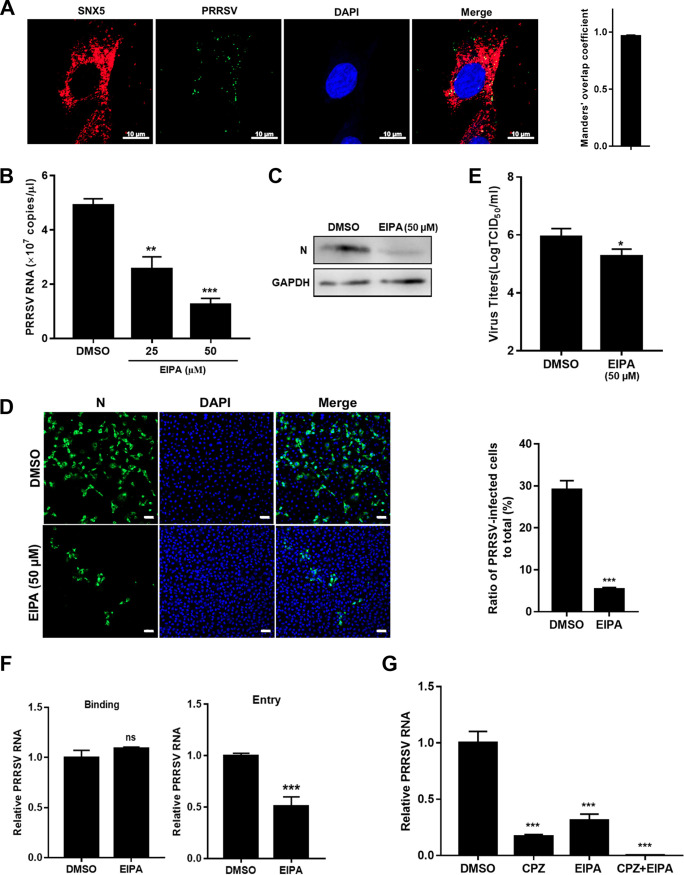
PRRSV utilizes macropinocytosis to infect MARC-145 cells. (A) Colocalization of PRRSV and SNX5-marked macropinosomes. MARC-145 cells were inoculated with MOI = 10 PRRSV at 37°C for 30 min. Cells were fixed and stained with anti-PRRSV N protein (green) and anti-SNX5 (red) antibody. Nuclei were stained with DAPI. Confocal microscopy was performed to detect the location. The colocalization was assessed by determination of Manders’ overlap coefficient. Scale bars, 10 μm. The addition of EIPA decreased PRRSV RNA abundance (B), N protein expression (C), infectivity (D), progeny viral titers (E), and entry (F). The serum-starved MARC-145 cells were pretreated with 25 μM EIPA, 50 μM EIPA, or DMSO and infected with PRRSV BJ-4 (MOI = 10) for 1 h. The cells were collected for assessment of PRRSV RNA abundance by RT-qPCR at 12 hpi, N protein expression by IB or immunofluorescence at 24 hpi, viral titers using TCID_50_ at 48 hpi, or binding and entry by RT-qPCR. Scale bars, 50 μm. (G) Simultaneous addition of EIPA and CPZ almost abolished PRRSV infection. The serum-starved MARC-145 cells were pretreated with 50 μM EIPA and/or 10 μM CPZ and then infected with PRRSV BJ-4 (MOI = 10) for 1 h. The cells were collected for assessment of PRRSV RNA abundance by RT-qPCR at 12 hpi. Each experiment was performed three times, and similar results were obtained. Differences between groups were assessed by using a Student *t* test, and the statistical significance is indicated (*, *P* < 0.05; **, *P* < 0.01; ***, *P* < 0.001; ns, not significant).

We analyzed whether PRRSV utilized macropinocytosis to infect MARC-145 cells. We first observed that internalized PRRSV virions colocalized with sorting nexin 5 (SNX5), a marker of specific endosomes for macropinocytosis (macropinosomes, Fig. 5A) (39). The colocalization coefficient was expressed as Manders’ overlap coefficient, and the value was >0.6, indicating an actual overlap of the signals and representing the true degree of colocalization (40). Ethylisopropyl amiloride (EIPA) specifically inhibits Na^+^/H^+^ exchanger activity and subsequent macropinocytosis (41). As shown in Fig. 5B to E, treatment with EIPA, compared to treatment with dimethyl sulfoxide (DMSO), led to 2- to 4-fold reductions in PRRSV RNA abundance, a 5-fold decrease in infectivity, and a 5-fold decrease in viral titers, respectively. Interestingly, the EIPA inhibited PRRSV infection during viral entry rather than binding (Fig. 5F). Since PRRSV infection was previously reported to be mediated by CME (22), we attempted to define the relative contribution of CME and macropinocytosis in PRRSV infection. We pretreated MARC-145 cells with chlorpromazine (CPZ), an inhibitor of clathrin lattice polymerization (42). Treatment with CPZ resulted in a greater decrease in PRRSV RNA abundance than treatment with EIPA (6.3-fold versus 3.5-fold). Simultaneous addition of these two inhibitors almost abolished PRRSV infection (Fig. 5G). All of these results indicate that, in addition to CME, PRRSV infects MARC-145 cells via macropinocytosis as an alternative pathway.

### Disruption of actin dynamics inhibits PRRSV infection via macropinocytosis.

**FIG 6:**
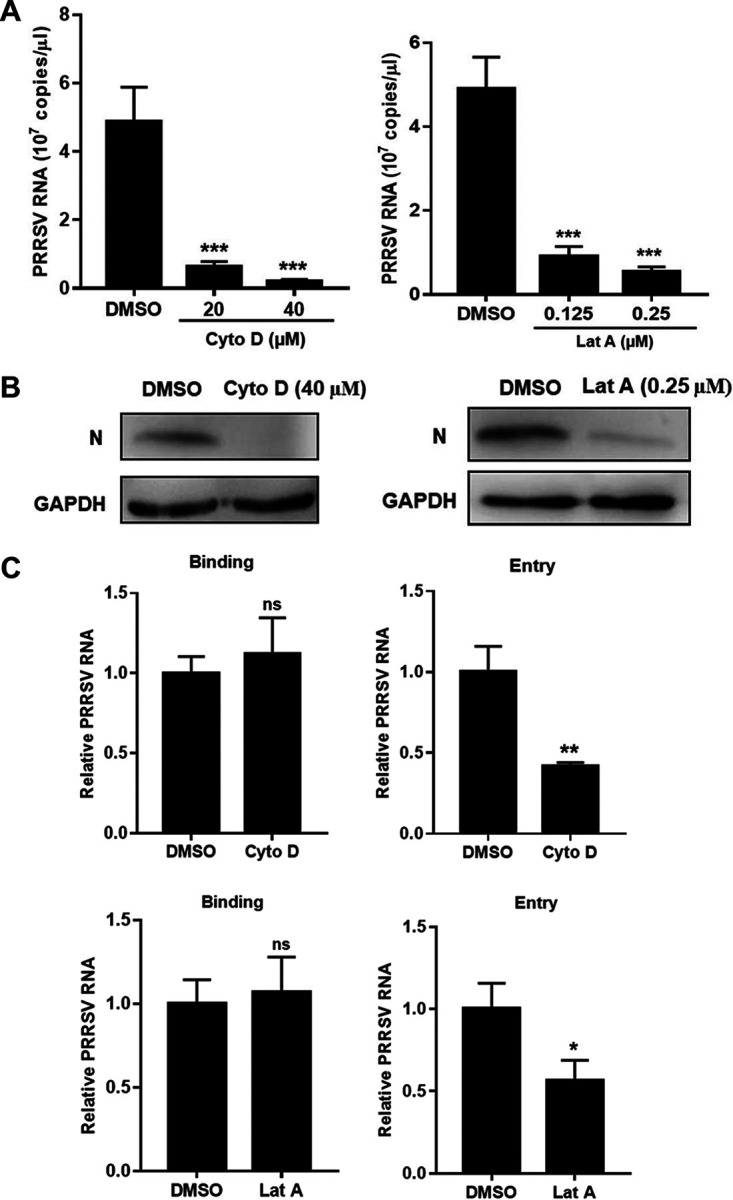
Disruption of actin dynamics inhibits PRRSV infection via macropinocytosis. Both Cyto D and Lat A decreased PRRSV RNA abundance (A), N protein expression (B), and entry (C). The serum-starved MARC-145 cells were pretreated with Cyto D (20 or 40 μM), Lat A (0.125 or 0.25 μM), or DMSO and infected with PRRSV BJ-4 (MOI = 10) for 1 h. The cells were collected for assessment of PRRSV RNA abundance by RT-qPCR at 12 hpi, N protein expression by IB at 24 hpi, or binding and entry by RT-qPCR. Each experiment was performed three times, and similar results were obtained. Differences between groups were assessed by using a Student *t* test, and the statistical significance is indicated (*, *P* < 0.05; **, *P* < 0.01; ***, *P* < 0.001; ns, not significant).

Since macropinocytosis requires actin rearrangements (9, 10), we explored whether the disruption of actin dynamics took effect on PRRSV infection. We preincubated MARC-145 cells with cytochalasin D (Cyto D) and latrunculin A (Lat A), respectively, and then inoculated with PRRSV. Cyto D disrupts actin microfilaments (43) and Lat A inhibits actin polymerization (44). As shown in Fig. 6, both inhibitors significantly suppressed PRRSV infection during viral entry in a dose-dependent manner.

### Rac1/Cdc42-Pak1 signaling pathway is involved in PRRSV infection via macropinocytosis.

**FIG 7:**
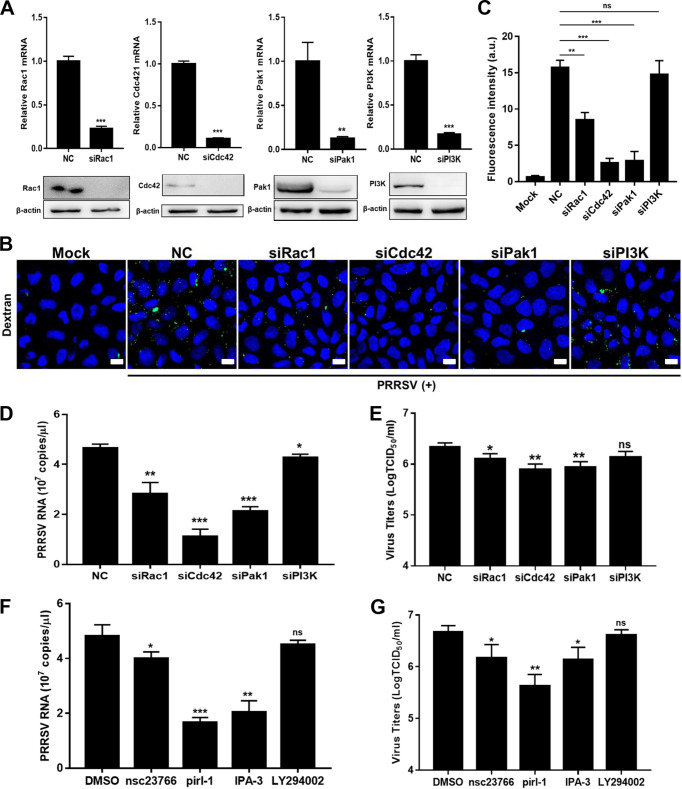
Rac1/Cdc42-Pak1 signaling pathway is involved in PRRSV infection via macropinocytosis. Knockdown of Rac1, Cdc42, and Pak1 (A) all decreased dextran uptake (B and C), PRRSV RNA abundance (D), and progeny viral titers (E). MARC-145 cells were transfected with siPak1, siRac1, siCdc42, siPI3K, or siRNA-NC for 36 h and infected with PRRSV BJ-4 (MOI = 10). The dextran uptake was detected at 30 mpi as stated above. Scale bars, 10 μm. The total fluorescence intensity of dextran was calculated using ImageJ software. The infected cells were collected to assess PRRSV RNA abundance by RT-qPCR at 12 hpi, and viral titers were determined from the TCID_50_ at 48 hpi. Inhibition of Rac1, Cdc42, and Pak1 all decreased PRRSV RNA abundance (F) and progeny viral titers (G). The serum-starved MARC-145 cells were pretreated with IPA-3 (10 μM), nsc23766 (50 μM), LY294002 (20 μM), pirl-1 (10 μM), or DMSO and infected with PRRSV BJ-4 (MOI = 10) for 1 h. The cells were collected for assessment of PRRSV RNA abundance by RT-qPCR at 12 hpi, and viral titers were determined from the TCID_50_ at 48 hpi. Each experiment was performed three times, and similar results were obtained. Differences between groups were assessed by using a Student *t* test, and the statistical significance is indicated (*, *P* < 0.05; **, *P* < 0.01; ***, *P* < 0.001; ns, not significant).

Another characteristic of macropinocytosis is its dependence on Rho GTPases, including Rac1 and cell division control protein 42 (Cdc42). A prominent downstream effect of these Rho GTPases is the activation of p21-activated kinase 1 (Pak1), which modulates actin cytoskeleton dynamics during macropinocytosis (9, 10). Therefore, we explored whether Rac1/Cdc42-Pak1 signaling pathway was involved in PRRSV infection via macropinocytosis. We first utilized specific small interference RNAs (siRNAs) targeting Rac1, Cdc42, and Pak1 (Fig. 7A). We found that knockdown of Rac1, Cdc42, and Pak1 inhibited macropinocytosis, as indicated by decreased dextran uptake (Fig. 7B and C). As shown in Fig. 7D and E, knockdown of Rac1, Cdc42, and Pak1 suppressed PRRSV infection. We further inhibited the activity of Rac1, Cdc42, or Pak1 with the selective chemical inhibitors nsc23766 (Rac1), pirl-1 (Cdc42), and IPA-3 (Pak1) and observed similar results (Fig. 7F and G). Phosphoinositol kinase-3 (PI3K) has been reported to be involved in multiple stages of macropinocytosis (9, 10). However, knockdown of PI3K did not suppress PRRSV-induced macropinocytosis in MARC-145 cells, and PRRSV infection was marginally influenced with treatment of siPI3K or its inhibitor LY294002 (Fig. 7). These results verify that Rac1/Cdc42-Pak1 signaling pathway is involved in PRRSV infection via macropinocytosis.

### PRRSV utilizes macropinocytosis to infect PAMs.

**FIG 8:**
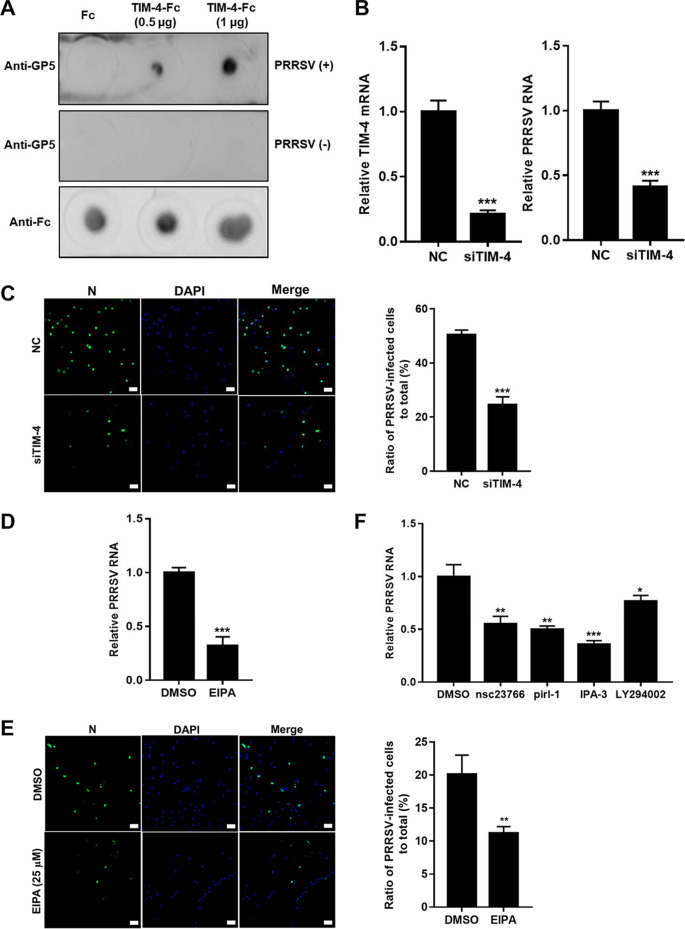
PRRSV utilizes macropinocytosis to infect PAMs. (A) TIM-4 directly binds to PRRSV, as demonstrated by dot blotting. TIM-4-Fc or Fc was spotted onto nitrocellulose membranes, followed by incubation with PRRSV BJ-4 for 4 h. Membranes were applied for PRRSV GP5 detection. *TIM-4* knockdown decreased PRRSV RNA abundance (B) and infectivity (C). PAMs were transfected with siTIM-4 or siRNA-NC for 36 h and infected with PRRSV BJ-4 (MOI = 10) for 1 h. The addition of EIPA decreased PRRSV RNA abundance (D) and infectivity (E). PAMs were pretreated with 25 μM EIPA or DMSO and infected with BJ-4 (MOI = 10) for 1 h. The cells were collected for assessment of PRRSV RNA abundance by RT-qPCR at 12 hpi, and N protein expression was determined by immunofluorescence at 24 hpi. (F) The Rac1/Cdc42-Pak1 signaling pathway was involved in PRRSV infection in PAMs. PAMs were pretreated with IPA-3 (5 μM), nsc23766 (25 μM), LY294002 (10 μM), pirl-1 (25 μM), or DMSO and infected with PRRSV BJ-4 (MOI = 10) for 1 h. The cells were collected for assessment of PRRSV RNA abundance by RT-qPCR at 12 hpi. Each experiment was performed three times, and similar results were obtained. Differences between groups were assessed by using a Student *t* test, and the statistical significance is indicated (*, *P* < 0.05; **, *P* < 0.01; ***, *P* < 0.001). Scale bars, 50 μm.

Since PAMs are primary *in vivo* target for PRRSV (19) and undergo constitutive macropinocytosis (45), we determined whether PRRSV utilizes this alternative pathway to infect the cells. TIM-4 is a homolog of TIM-1 specially expressed in macrophages (7). We determined that TIM-4 directly bound to PRRSV as TIM-1 (Fig. 8A). *TIM-4* knockdown played a significant inhibitory effect on PRRSV infection (2- to 2.5-fold, Fig. 8B and C). The impacts of EIPA on PRRSV infection were also demonstrated (Fig. 8D and E). The Rac1/Cdc42-Pak1 signaling pathway was involved in PRRSV infection in PAMs as well (Fig. 8F). These results show that macropinocytosis is utilized by PRRSV in both PAMs and MARC-145 cells.

### CD163 is essential for PRRSV infection via TIM-induced macropinocytosis.

**FIG 9:**
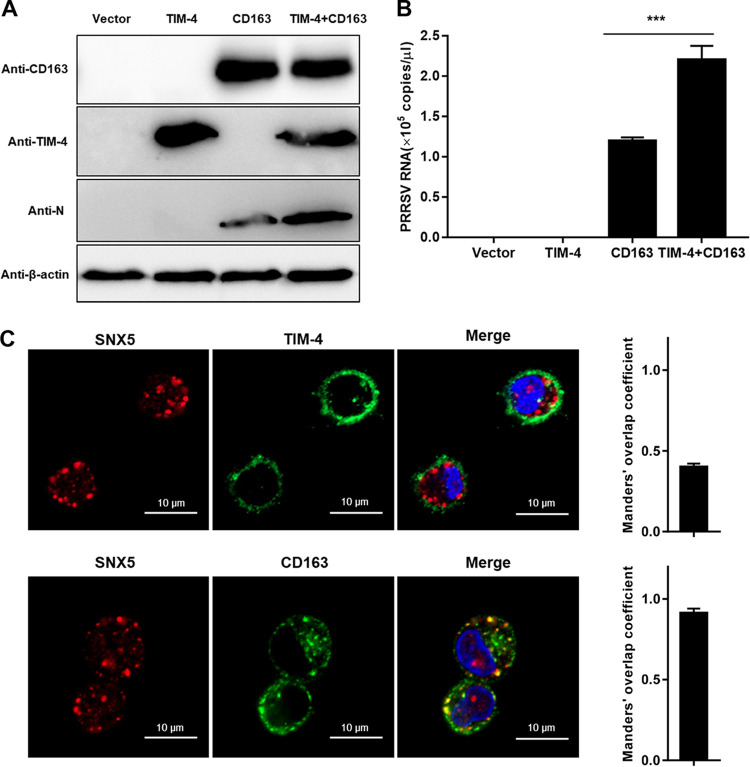
CD163 is essential for PRRSV infection via macropinocytosis. (A and B) CD163 is required for PRRSV infection via macropinocytosis. BHK-21 cells were transfected with TIM-4 and/or CD163 for 36 h and then infected with PRRSV BJ-4 (MOI = 10) for 1 h. RT-qPCR and IB were performed with specific primers and antibodies at 12 and 24 hpi, respectively. (C) Colocalization of PRRSV and CD163 in SNX5-marked macropinosomes. PAMs were inoculated with an MOI of 10 PRRSV BJ-4 at 37°C for 30 min. Cells were fixed and stained with anti-SNX5 antibody and anti-TIM4 or anti-CD163 antibody. Nuclei were stained with DAPI, followed by confocal microscopy. The colocalization was assessed by determination of the Manders’ overlap coefficient. Each experiment was performed three times, and similar results were obtained. Scale bars, 10 μm.

It is well established that CD163 is an indispensable receptor for PRRSV infection (24, 27). We considered the involvement of CD163 and TIMs in PRRSV infection via macropinocytosis. Figure 9A and B show that expression of TIM-4 alone in baby hamster kidney 21 (BHK-21) cells was not sufficient to support PRRSV infection. In contrast, the expression of CD163 alone conferred susceptibility to PRRSV infection, consistent with a previous study (24). Importantly, the coexpression of TIM-4 and CD163 contributed to PRRSV infection more than did the expression of CD163 alone. Furthermore, CD163 colocalized with PRRSV in SNX5-marked macropinosomes in PAMs, whereas most TIM-4s did not (Fig. 9C). Consequently, CD163 is essential during PRRSV infection via TIM-induced macropinocytosis.

## DISCUSSION

Viruses usually exploit multiple strategies to infect host cells and establish infection (1, 2, 35). Viral apoptotic mimicry has been documented for many enveloped viruses to facilitate viral infections, including alphaviruses, flaviviruses, filoviruses, some arenaviruses, baculoviruses, poxviruses, and rhabdoviruses (46–49). Whether arteriviruses use viral apoptotic mimicry has not yet been authenticated. In the present study, we demonstrated that PRRSV utilized viral apoptotic mimicry and TIM-induced macropinocytosis to promote infection for the first time.

Several PSRs, including TIM-1/4, have been identified to enhance virus infection (7). In particular, TIMs serve as entry factors or even receptors for dengue virus (29) and Ebola virus (50, 51). It is also worth noting that not all PSRs enhance viral entry. For example, the PSRs Stabilin-1/2 and BAI1 do not appear to enhance viral entry (52). Here, we determined that TIM-1/4 interacted with PRRSV virions (Fig. 3 and 8) and induced macropinocytosis (Fig. 4) upon viral infection, whereas Axl did not (Fig. 2). However, TIM-4 was not sufficient to support PRRSV infection and internalized into macropinosomes in PAMs as CD163 (Fig. 9). Consequently, we assume that TIM-1/4 might only function as an attachment factor for PRRSV and inducer of downstream signaling of macropinocytosis. The detailed mechanisms involved in TIM-PRRSV interaction and TIM induction need to be investigated.

Macropinocytosis is usually induced by external stimuli, which may be associated with growth factors-triggered activation of receptor tyrosine kinases (9). Among them, epidermal growth factor receptor (EGFR) has been demonstrated to induce macropinocytosis (53). Although the EGFR-PI3K signaling pathway has been recently reported to be required for actin reorganization and efficient PRRSV entry into MARC-145 cells (54, 55), whether PRRSV induces and utilizes macropinocytosis to infect host cells has not been clarified.

To exclude the interference of growth factors and EGFR, we utilized purified PRRSV virions and serum-starved cells throughout our research unless stated otherwise. Here, we found that PRRSV strains externalized PS on their envelopes as viral apoptotic mimicry (Fig. 1). The specific mechanisms by which PRRSV incorporates and exposes PS are our next issue to be studied. Subsequently, we identified that PRRSV is recognized as apoptotic mimicry by TIM-1/4 (Fig. 2, 3, and 8). Moreover, we determined that PRRSV induced macropinocytosis via TIM-1 in MARC-145 cells and utilized the pathway to infect both MARC-145 cells and PAMs (Fig. 4, 5, and 8). All of these results concluded that PRRSV directly induces macropinocytosis via TIM and infects host cells via the pathway. It would be interesting to investigate whether PRRSV exploits other strategies to induce macropinocytosis. We tried to address the individual contribution of macropinocytosis and CME to PRRSV infection and found that CME might contribute more than macropinocytosis during PRRSV infection (Fig. 5). It would be meaningful to define this contribution under actual *in vivo* conditions in the future.

The Rac1/Cdc42-Pak1 signaling pathway was determined to mediate PRRSV infection via macropinocytosis, whereas PI3K was shown to be minimally involved (Fig. 7). We speculated that PI3K might be involved in external stimulus (e.g., EGF-EGFR)-induced macropinocytosis instead of PRRSV-induced macropinocytosis via TIMs. The discrepancy with previous studies should be addressed (55–57).

Other factors, including protein kinase C (PKC) (58) and myosin II (59), are also responsible for macropinocytosis. A recent work has demonstrated that PKC is beneficial to PRRSV replication and infection (60). In addition, nonmuscle myosin IIA, encoded by *myosin heavy chain 9* (*MYH9*) is an essential factor for PRRSV infection (61). Mechanistically, blebbistatin, an inhibitor of myosin II heavy chain activity (62), impairs the viral entry (61). All of these reports strengthen our conclusion that PRRSV utilizes macropinocytosis to infect host cells.

**FIG 10:**
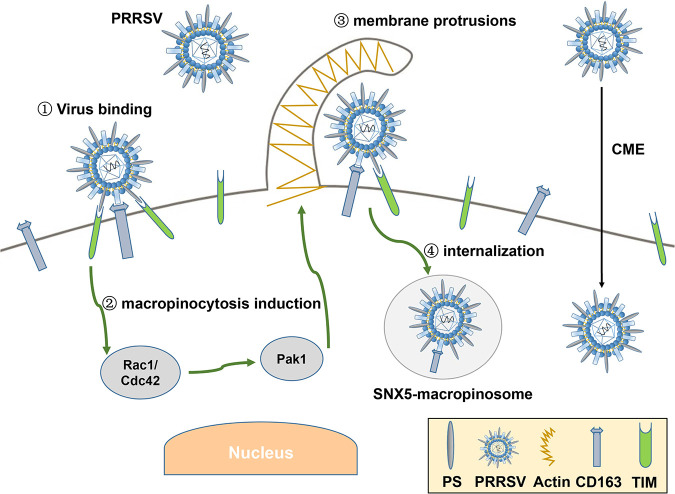
Model showing how PRRSV utilizes viral apoptotic mimicry and TIM-induced macropinocytosis as an alternative pathway to infect host cells. In addition to CME, TIM-1/4 recognizes PRRSV as apoptotic mimicry and induces macropinocytosis via the Rac1/Cdc42-Pak1 signaling pathway. PRRSV enters SNX5-macropinosomes via macropinocytosis as an alternative pathway, where CD163 functions as an indispensable receptor.

Based on the results stated above, we propose a model to depict PRRSV infection (Fig. 10). In addition to CME, TIM-1/4 recognizes PRRSV as apoptotic mimicry and induces macropinocytosis via the Rac1/Cdc42-Pak1 signaling pathway. PRRSV enters SNX5-macropinosomes via macropinocytosis as an alternative pathway, where CD163 functions as an indispensable receptor.

In conclusion, we demonstrate that PRRSV utilizes viral apoptotic mimicry and TIM-induced macropinocytosis as an alternative pathway to infect host cells. The results we obtained deepen our understanding of PRRSV complicated infection and provide novel opportunities for the development of drugs and vaccines against PRRSV.

## MATERIALS AND METHODS

### Cells and viruses.

PAMs were prepared from lung lavage of 4-week-old specific-pathogen-free pigs. The experimental procedure for the collection of PAMs was authorized and supervised by the Ethical and Animal Welfare Committee of Key Laboratory of Animal Immunology of the Ministry of Agriculture of China (permit 2017008). PAMs were maintained in Roswell Park Memorial Institute 1640 medium (RPMI 1640) supplemented with 10% heat-inactivated fetal bovine serum (FBS; Gibco, Carlsbad, CA), penicillin (100 U/ml; Gibco), and streptomycin (100 mg/ml; Gibco) in a humidified 37°C, 5% CO_2_ incubator. MARC-145 and BHK-21 cells were purchased from Cellbio (Shanghai, China) and maintained in Dulbecco modified Eagle medium supplemented with 10% heat-inactivated FBS and penicillin-streptomycin.

HP-PRRSV strain HN07-1 (GenBank accession number KX766378.1) was previously isolated by our laboratory (63). A typical PRRSV-2 strain, BJ-4 (GenBank accession number AF331831), and PRRSV-1 strain GZ11-G1 (GenBank accession number KF001144) were kindly provided by Hanchun Yang from China Agricultural University (64). PRRSV virions were purified by sucrose density gradient ultracentrifugation as previously described (65), and their infectivities were comparable to those of naive virions (data not shown). Purified PRRSV virions in phosphate-buffered saline (PBS) were utilized throughout this work.

### Antibodies, inhibitors, and reagents.

The antibodies used were anti-TIM-1 rabbit polyclonal antibody (catalog no. ab47635), anti-human IgG antibody (EPR4421; catalog no. ab109489), anti-TIM-4 antibody (catalog no. ab47636), anti-SNX5 antibody (EPR14358; catalog no. ab180520), horseradish peroxidase (HRP)-labeled goat anti-rabbit IgG antibody (catalog no. ab6721), and HRP-labeled goat anti-mouse IgG antibody (catalog no. ab6789), all purchased from Abcam (Cambridge, United Kingdom). β-Actin (8H10D10) mouse monoclonal antibody (MAb; catalog no. 3700), Axl (C89E7) rabbit MAb (catalog no. 8661), PI3 kinase p110α (C73F8) rabbit MAb (catalog no. 4249), PAK1 antibody (catalog no. 2602), Rac1/2/3 antibody (catalog no. 2465), and Cdc42 antibody (catalog no. 2462) were all purchased from Cell Signaling Technology (Danvers, CT). Anti-PS mouse MAb 1H6 (catalog no. 05-719) and isotype control mouse IgG (catalog no. NI03) were purchased from Merck Millipore (Ontario, Canada). CD163 antibody 2A10/11 (catalog no. MCA2311GA) and CD163 antibody EDHu-1 (catalog no. MCA1853) were purchased from Bio-Rad Antibodies (Hercules, CA). TIM-4 antibody (catalog no. sc390805) was purchased from Santa Cruz Biotechnology (Santa Cruz, CA). Mouse MAbs against PRRSV N protein and GP5 were kept in our laboratory (30). Alexa Fluor 488-goat anti-mouse antibody (catalog no. A-11029), Alexa Fluor 647-goat anti-mouse antibody (catalog no. A-21235), and Alexa Fluor 647-goat anti-rabbit antibody (catalog no. A-21245) were purchased from Invitrogen (Carlsbad, CA).

The inhibitors CPZ (catalog no. c0982), Lat A (catalog no. 428021), IPA-3 (catalog no. I2285), nsc23766 (catalog no. SML0952), and LY294002 (catalog no. 19-142) were all purchased from Sigma-Aldrich (St. Louis, MO). EIPA (catalog no. sc-202458) was purchased from Santa Cruz Biotechnology. Cyto D (catalog no. PHZ1063) was purchased from Invitrogen. Pirl-1 (catalog no. 5137877) was purchased from ChemBridge (San Diego, CA).

The reagents Alexa Fluor 488-phalloidin (catalog no. A12379), Alexa Fluor 647-transferrin (catalog no. T23366), annexin V-conjugated Alexa Fluor 488 (catalog no. A13201), annexin-binding buffer for flow cytometry (catalog no. v13246), ProLong glass antifade mountant (catalog no. P36984), Lipofectamine LTX with Plus reagent (catalog no. 15338030), and Lipofectamine RNAiMAX transfection reagent (catalog no. 13778150) were purchased from Invitrogen. The CellTiter 96 AQueous One Solution cell proliferation assay (MTS; catalog no. G3582) was purchased from Promega (Madison, WI). Recombinant TIM-1-Fc (catalog no. SRP8054), recombinant TIM-4-Fc (catalog no. SRP8057), and fluorescein isothiocyanate (FITC)-dextran (average molecular weight, 70,000; catalog no. 46945) were purchased from Sigma-Aldrich.

### PS detection.

For detection of PS on PRRSV envelope, MARC-145 cells were washed with PBS and dissociated by using an enzyme-free cell dissociation solution (catalog no. 13151014; Gibco). The cells were then collected at 4°C for 1 h and inoculated with virions at a multiplicity of infection (MOI) of 10 or with PBS as an unbound control. The cells were washed three times with cold PBS, resuspended in annexin-binding buffer, and incubated with annexin V-conjugated Alexa Fluor 488 at 4°C for 20 min. After incubation, the cells were added with annexin-binding buffer, mixed gently, and kept on ice. PS detection was preceded with FCM immediately.

Purified virions in PBS were spotted onto nitrocellulose membranes (Pierce, Rockford, IL), and PBS was spotted onto membranes as a negative control. Membranes were dried and blocked with 5% bovine serum albumin (BSA) in PBS at 4°C overnight. Next, the membranes were incubated with the anti-PS MAb or anti-PRRSV GP5 MAb as a viral loading control for 1 h at 37°C. After three washes with PBS plus Tween 20 (PBST), the membranes were incubated with HRP-conjugated goat anti-mouse IgG antibody and detected by enhanced chemiluminescence (ECL) Plus reagent (Solarbio, Beijing, China).

### Detection of PSR transcription.

MARC-145 cells were collected, and the total RNAs were extracted using TRIzol reagent (Invitrogen). The reverse transcription cDNAs were prepared by using a PrimeScript RT reagent kit with gDNA Eraser (TaKaRa, Dalian, China) according to the manufacturer’s instructions. The cDNAs were then subjected to PCR with specific primers for TIM-1, Axl, Stabilin-1, and Stabilin-2 (Table 1). The PCR products were subjected to agarose gel electrophoresis.

**TABLE 1:** Primers used in this study

Target gene	Sequence (5′–3′)	
	Sense	Antisense
PRRSV-ORF7	AAACCAGTCCAGAGGCAAGG	GCAAACTAAACTCCACAGTGTAA
Monkey TIM-1	ACCTTTGTTCCTCCAACGCC	CAGCAGTGTCATAGGGTGGG
Pig TIM-4	GTCGGTGACTTTGCCCTGTA	TTGGCTGACTTCCTCGACAC
Monkey Axl	GGGAGATTGCCACAAGAG	GTGACATCAAGGCATACA
Monkey Pak1	TTGACCCGGAATACTGAGA	TGAAGCACCTTGTCCAATC
Monkey Rac1	CAGTGTTTGACGAAGCGA	CAAGGGACAGGACCAAGA
Monkey Cdc42	CAGATTACGACCGCTGAGT	AGGCACCCACTTTTCTTTC
Monkey PI3K	CTTCCACACAATTAAACAGCA	ATTCCTATGCAATCGGTCTT
Monkey Stabilin-1	GCGATGGGATAGTGTGT	CATTGCTGTTGATGCTGAC
Monkey Stabilin-2	GGACCAGGATGAGAAAAGC	TGCCAAGTGAAGGAAGTTG
GAPDH	CCTTCCGTGTCCCTACTGCCAAC	GACGCCTGCTTCACCACCTTCT

### RT-qPCR.

Total RNAs were extracted using TRIzol reagent, and the reverse transcription cDNAs were prepared as described above. Then, RT-qPCR was performed using FastStart Universal SYBR green Master (Rox, catalog no. 4913850001; Roche, Basel, Switzerland) on a 7500 Fast RT-PCR system (Applied Biosystems, Foster City, CA). A plasmid containing PRRSV ORF7 was used as the template to generate a standard curve, and the actual viral RNA copies were calculated based on this curve (66). The relative RNA level was evaluated by the 2^–ΔΔ^*^CT^* method using glyceraldehyde-3-phosphate dehydrogenase (GAPDH) mRNA as an endogenous control (67). The primers for RT-qPCR are listed in Table 1.

### IB.

Cells were harvested and lysed in radioimmunoprecipitation assay lysis buffer (Beyotime Biotechnology, Shanghai, China) supplemented with a cocktail of protease inhibitors (Roche). The lysates were separated by 10% to 15% gradient sodium dodecyl sulfate-polyacrylamide gel electrophoresis (SDS-PAGE), and electrotransferred onto polyvinylidene fluoride membranes (Merck Millipore). The membranes were blocked in 5% skim milk for 1 h and probed with the indicated primary antibodies. After incubation with HRP-labeled goat anti-mouse or rabbit IgG antibody as a secondary antibody, the indicated proteins were detected by ECL Plus reagent.

### RNA interference.

All siRNAs and siRNA negative controls (siRNA-NC) were designed and synthesized by GenePharma (Shanghai, China). In knockdown experiments, PAMs or MARC-145 cells were transfected with the indicated siRNAs at a final concentration of 10 nM using Lipofectamine RNAiMAX according to the manufacturer’s instructions for 36 h. After the cell viability was measured by the CellTiter 96 AQueous One Solution assay kit (data not shown), transfected cells were applied for subsequent experiments. The indicated siRNAs are listed in Table 2.

**TABLE 2:** siRNAs used in this study

Target gene	Sequence (5′–3′)	
	Sense	Antisense
Monkey TIM-1	GCUCACCAUUGUACUCUUATT	UAAGAGUACAAUGGUGAGCTT
Monkey Axl	CCUGUGGUCAUCUUACCUUTT	AAGGUAAGAUGACCACAGGTT
Monkey Rac1	CCUAGUGGGAACUAAACUUTT	AAGUUUAGUUCCCACUAGGTT
Monkey Cdc42	AGACUCCUUUCUUGCUUGUTT	ACAAGCAAGAAAGGAGUCUTT
Monkey Pak1	CCACUCCACCAGAUGCUUUTT	AAAGCAUCUGGUGGAGUGGTT
Monkey PI3K	CCACACAAUUAAACAGCAUTT	AUGCUGUUUAAUUGUGUGGTT
Pig TIM-4	CCCGUGUCCCAAAUCCAAATT	UUUGGAUUUGGGACACGGGTT
siRNA-NC	UUCUCCGAACGUGUCACGUTT	ACGUGACACGUUCGGAGAATT

### Virus titration assay.

The treated cells were inoculated with PRRSV at an MOI of 10 and incubated at 37°C for 3 h. The viruses not entering the cells were then washed away. At 48 hpi, the viral yields were measured by determining a 50% tissue culture infected dose (TCID_50_) assay in MARC-145 cells (68).

### Binding and entry assay.

Cells were serum starved for 1 h at 37°C and inoculated with purified virions at an MOI of 10 for 1 h at 4°C, allowing for viral attachment without internalization. The cells were then washed with cold PBS three times so that unbound viruses were removed. The culture medium was replaced with fresh serum-free medium, and the cells were subsequently shifted to 37°C, allowing virus internalization. After 1 h, the cells were washed with citrate buffer solution (pH 3.0) to remove the noninternalized visions and washed with PBS three times. PRRSV RNA abundance was determined by RT-qPCR.

### Pulldown assay.

The recombinant Fc-fused TIM-1/4 was first bound to protein A/G-beads (Pierce) at 4°C for 4 h. PRRSV virions were subsequently incubated with the beads at 4°C overnight. After extensive washing with PBS, the target proteins were eluted and subjected to IB with the indicated antibodies.

### Dot blot assay.

TIM-1/4-Fc proteins were spotted onto nitrocellulose membranes. Membranes were dried and blocked with 5% BSA in PBS 4°C overnight. Next, membranes were incubated with the PRRSV virions at 4°C for 4 h. After three washes with PBST, the membranes were incubated with the indicated primary antibodies and detected with HRP-conjugated antibodies.

### Confocal microscopy.

Cells were grown in 24-well plates on glass coverslips, fixed with 4% paraformaldehyde (PFA) for 15 min, and permeabilized with 0.1% Triton X-100 in PBS for 5 min at room temperature. Phalloidin (dilution 1:100) or DAPI (4′,6′-diamidino-2-phenylindole) was used to stain actin filaments and nuclei, respectively. Alternatively, cells were stained with the appropriate primary and secondary antibodies. Coverslips were mounted to the glass slides and examined by using a microscope (LSM700; Carl Zeiss AG, Oberkochen, Germany) with the confocal laser scanning setup (20×, 40×, or 63× objective). The numerical aperture (NA) of the 20× objective is 0.8, the NA of the 40× objective is 0.95, and the NA of the 63× objective is 1.4. Images were representative as a single slice of a stack from three independent experiments (69). Colocalization analyses were carried out according to the method of Zinchuk and Grossenbacher-Zinchuk (40). Manders’ overlap coefficient (>0.6) shows an actual overlap of the signals and is considered to represent the true degree of colocalization. Quantitative analyses of single-channel fluorescence were performed using ImageJ software (70, 71).

### FITC-dextran uptake.

MARC-145 cells were grown in 24-well plates on glass coverslips. Prior to FITC-dextran uptake, the cells were serum starved for 2 h. FITC-dextran was incubated with the cells (final concentration, 0.25 mg/ml) in the absence or presence of PRRSV virions at an MOI of 10 for different time periods (15, 30, and 45 min). The cells were then washed three times with cold PBS on ice and once with low-pH buffer (0.1 M sodium acetate, 0.05 M NaCl [pH 5.5]), fixed with 4% PFA in PBS, and subsequently permeabilized with 0.1% Triton X-100 in PBS. After mounting, the slides were examined by confocal microscopy.

### Cytotoxicity of inhibitors.

MARC-145 cells or PAMs were seeded onto 96-well plates and pretreated with indicated inhibitors at 37°C for 4 h. The cell viability was then measured using a CellTiter 96 AQueous One Solution cell proliferation assay. Briefly, the assay was performed by adding CellTiter 96 AQueous One Solution reagent to wells, followed by incubation for 4 h. The absorbance at 490 nm was then recorded with a Bio-Rad microplate reader (data not shown).

### Inhibitor treatments.

MARC-145 cells and PAMs were serum starved for 1 h and treated with noncytotoxic specific inhibitors or DMSO for 1 h at 37°C in serum-free medium before subsequent experiments.

### Plasmid transfection.

The optimized cDNA encoding porcine TIM-4 was cloned into the vector pECMV-MCS-FLAG (kept in our laboratory). A construct with complete porcine CD163 cDNA integrated into the PiggyBac transposon system was kindly provided by Enmin Zhou (Northwest Agriculture and Forestry University, China) (72). BHK-21 cells were seeded at a density of 4 × 10^5^ cells/ml culture medium overnight. The cells were then transfected with TIM-4 and/or CD163 plasmid using Lipofectamine LTX with Plus reagent according to the manufacturer’s instructions. The protein expression was tested by IB as stated above.

### Statistical analysis.

Three replicates were included in all experiments, and each experiment was independently performed three times. The experimental data are presented as group means and standard deviations (SD) and were analyzed by the unpaired two-tailed Student *t* test with GraphPad Prism software (v7.0). Statistical significance is indicated in the figures (*, *P* < 0.05; **, *P* < 0.01; ***, *P* < 0.001; ns, not significant).

## References

[1] Cossart P, Helenius A 2014 Endocytosis of viruses and bacteria. Cold Spring Harb Perspect Biol 6:a016972. doi:10.1101/cshperspect.a016972. 25085912 PMC4107984

[2] Helenius A 2018 Virus entry: looking back and moving forward. J Mol Biol 430:1853–1862. doi:10.1016/j.jmb.2018.03.034. 29709571 PMC7094621

[3] Segawa K, Nagata S 2015 An apoptotic “eat me” signal: phosphatidylserine exposure. Trends Cell Biol 25:639–650. doi:10.1016/j.tcb.2015.08.003. 26437594

[4] Hoffmann PR, deCathelineau AM, Ogden CA, Leverrier Y, Bratton DL, Daleke DL, Ridley AJ, Fadok VA, Henson PM 2001 Phosphatidylserine (PS) induces PS receptor-mediated macropinocytosis and promotes clearance of apoptotic cells. J Cell Biol 155:649–659. doi:10.1083/jcb.200108080. 11706053 PMC2198875

[5] Li MO, Sarkisian MR, Mehal WZ, Rakic P, Flavell RA 2003 Phosphatidylserine receptor is required for clearance of apoptotic cells. Science 302:1560–1563. doi:10.1126/science.1087621. 14645847

[6] Amara A, Mercer J 2015 Viral apoptotic mimicry. Nat Rev Microbiol 13:461–469. doi:10.1038/nrmicro3469. 26052667 PMC7097103

[7] Moller-Tank S, Maury W 2014 Phosphatidylserine receptors: enhancers of enveloped virus entry and infection. Virology 468-470:565–580. doi:10.1016/j.virol.2014.09.009. 25277499 PMC4252826

[8] Marsh M, McMahon HT 1999 The structural era of endocytosis. Science 285:215–220. doi:10.1126/science.285.5425.215. 10398591

[9] Mercer J, Helenius A 2009 Virus entry by macropinocytosis. Nat Cell Biol 11:510–520. doi:10.1038/ncb0509-510. 19404330

[10] Mercer J, Helenius A 2012 Gulping rather than sipping: macropinocytosis as a way of virus entry. Curr Opin Microbiol 15:490–499. doi:10.1016/j.mib.2012.05.016. 22749376

[11] Rossow KD 1998 Porcine reproductive and respiratory syndrome. Vet Pathol 35:1–20. doi:10.1177/030098589803500101. 9545131

[12] Lunney JK, Benfield DA, Rowland RR 2010 Porcine reproductive and respiratory syndrome virus: an update on an emerging and re-emerging viral disease of swine. Virus Res 154:1–6. doi:10.1016/j.virusres.2010.10.009. 20951175 PMC7172856

[13] Holtkamp DJ, Kliebenstein JB, Neumann EJ 2013 Assessment of the economic impact of porcine reproductive and respiratory syndrome virus on United States pork producers. J Swine Health Prod 21:72–84.

[14] Done SH, Paton DJ 1995 Porcine reproductive and respiratory syndrome: clinical disease, pathology and immunosuppression. Vet Rec 136:32–35. doi:10.1136/vr.136.2.32. 7709569

[15] Snijder EJ, Meulenberg JJ 1998 The molecular biology of arteriviruses. J Gen Virol 79:961–979. doi:10.1099/0022-1317-79-5-961. 9603311

[16] Snijder EJ, Kikkert M, Fang Y 2013 Arterivirus molecular biology and pathogenesis. J Gen Virol 94:2141–2163. doi:10.1099/vir.0.056341-0. 23939974

[17] Shi M, Lam TT, Hon CC, Hui RK, Faaberg KS, Wennblom T, Murtaugh MP, Stadejek T, Leung FC 2010 Molecular epidemiology of PRRSV: a phylogenetic perspective. Virus Res 154:7–17. doi:10.1016/j.virusres.2010.08.014. 20837072

[18] Adams MJ, Lefkowitz EJ, King AMQ, Harrach B, Harrison RL, Knowles NJ, Kropinski AM, Krupovic M, Kuhn JH, Mushegian AR, Nibert M, Sabanadzovic S, Sanfacon H, Siddell SG, Simmonds P, Varsani A, Zerbini FM, Gorbalenya AE, Davison AJ 2017 Changes to taxonomy and the International Code of Virus Classification and Nomenclature ratified by the International Committee on Taxonomy of Viruses (2017). Arch Virol 162:2505–2538. doi:10.1007/s00705-017-3358-5. 28434098

[19] Duan X, Nauwynck HJ, Pensaert MB 1997 Virus quantification and identification of cellular targets in the lungs and lymphoid tissues of pigs at different time intervals after inoculation with porcine reproductive and respiratory syndrome virus (PRRSV). Vet Microbiol 56:9–19. doi:10.1016/S0378-1135(96)01347-8. 9228678

[20] Kim HS, Kwang J, Yoon IJ, Joo HS, Frey ML 1993 Enhanced replication of porcine reproductive and respiratory syndrome (PRRS) virus in a homogeneous subpopulation of MA-104 cell line. Arch Virol 133:477–483. doi:10.1007/BF01313785. 8257302

[21] Kreutz LC, Ackermann MR 1996 Porcine reproductive and respiratory syndrome virus enters cells through a low-pH-dependent endocytic pathway. Virus Res 42:137–147. doi:10.1016/0168-1702(96)01313-5. 8806181

[22] Nauwynck HJ, Duan X, Favoreel HW, Van Oostveldt P, Pensaert MB 1999 Entry of porcine reproductive and respiratory syndrome virus into porcine alveolar macrophages via receptor-mediated endocytosis. J Gen Virol 80:297–305. doi:10.1099/0022-1317-80-2-297. 10073688

[23] Van Breedam W, Delputte PL, Van Gorp H, Misinzo G, Vanderheijden N, Duan X, Nauwynck HJ 2010 Porcine reproductive and respiratory syndrome virus entry into the porcine macrophage. J Gen Virol 91:1659–1667. doi:10.1099/vir.0.020503-0. 20410315

[24] Calvert JG, Slade DE, Shields SL, Jolie R, Mannan RM, Ankenbauer RG, Welch SK 2007 CD163 expression confers susceptibility to porcine reproductive and respiratory syndrome viruses. J Virol 81:7371–7379. doi:10.1128/JVI.00513-07. 17494075 PMC1933360

[25] Shi C, Liu Y, Ding Y, Zhang Y, Zhang J 2015 PRRSV receptors and their roles in virus infection. Arch Microbiol 197:503–512. doi:10.1007/s00203-015-1088-1. 25666932

[26] Zhang Q, Yoo D 2015 PRRS virus receptors and their role for pathogenesis. Vet Microbiol 177:229–241. doi:10.1016/j.vetmic.2015.04.002. 25912022

[27] Whitworth KM, Rowland RR, Ewen CL, Trible BR, Kerrigan MA, Cino-Ozuna AG, Samuel MS, Lightner JE, McLaren DG, Mileham AJ, Wells KD, Prather RS 2016 Gene-edited pigs are protected from porcine reproductive and respiratory syndrome virus. Nat Biotechnol 34:20–22. doi:10.1038/nbt.3434. 26641533

[28] Walker JH, Boustead CM, Koster JJ, Bewley M, Waller DA 1992 Annexin V, a calcium-dependent phospholipid-binding protein. Biochem Soc Trans 20:828–833. doi:10.1042/bst0200828. 1487073

[29] Meertens L, Carnec X, Lecoin MP, Ramdasi R, Guivel-Benhassine F, Lew E, Lemke G, Schwartz O, Amara A 2012 The TIM and TAM families of phosphatidylserine receptors mediate dengue virus entry. Cell Host Microbe 12:544–557. doi:10.1016/j.chom.2012.08.009. 23084921 PMC3572209

[30] Liu Y, Li R, Chen XX, Zhi Y, Deng R, Zhou EM, Qiao S, Zhang G 2019 Nonmuscle myosin heavy chain IIA recognizes sialic acids on sialylated RNA viruses to suppress proinflammatory responses via the DAP12-Syk pathway. mBio 10:e00574-19. doi:10.1128/mBio.00574-19. 31064828 PMC6509187

[31] Guo Z, Chen XX, Li R, Qiao S, Zhang G 2018 The prevalent status and genetic diversity of porcine reproductive and respiratory syndrome virus in China: a molecular epidemiological perspective. Virol J 15:2. doi:10.1186/s12985-017-0910-6. 29301547 PMC5753475

[32] Song J, Gao P, Kong C, Zhou L, Ge X, Guo X, Han J, Yang H 2019 The nsp2 hypervariable region of porcine reproductive and respiratory syndrome virus strain JXwn06 is associated with viral cellular tropism to primary porcine alveolar macrophages. J Virol 93:e01436-19. doi:10.1128/JVI.01436-19. PMC6880165 31554681

[33] Gao P, Chai Y, Song J, Liu T, Chen P, Zhou L, Ge X, Guo X, Han J, Yang H 2019 Reprogramming the unfolded protein response for replication by porcine reproductive and respiratory syndrome virus. PLoS Pathog 15:e1008169. doi:10.1371/journal.ppat.1008169. 31738790 PMC6932825

[34] Doherty GJ, McMahon HT 2009 Mechanisms of endocytosis. Annu Rev Biochem 78:857–902. doi:10.1146/annurev.biochem.78.081307.110540. 19317650

[35] Mercer J, Schelhaas M, Helenius A 2010 Virus entry by endocytosis. Annu Rev Biochem 79:803–833. doi:10.1146/annurev-biochem-060208-104626. 20196649

[36] Conner SD, Schmid SL 2003 Regulated portals of entry into the cell. Nature 422:37–44. doi:10.1038/nature01451. 12621426

[37] Jones AT 2007 Macropinocytosis: searching for an endocytic identity and role in the uptake of cell penetrating peptides. J Cell Mol Med 11:670–684. doi:10.1111/j.1582-4934.2007.00062.x. 17760832 PMC3823249

[38] Dancker P, Low I, Hasselbach W, Wieland T 1975 Interaction of actin with phalloidin: polymerization and stabilization of F-actin. Biochim Biophys Acta 400:407–414. doi:10.1016/0005-2795(75)90196-8. 126084

[39] Lim JP, Wang JTH, Kerr MC, Teasdale RD, Gleeson PA 2008 A role for SNX5 in the regulation of macropinocytosis. BMC Cell Biol 9:58–58. doi:10.1186/1471-2121-9-58. 18854019 PMC2576169

[40] Zinchuk V, Grossenbacher-Zinchuk O 2009 Recent advances in quantitative colocalization analysis: focus on neuroscience. Prog Histochem Cytochem 44:125–172. doi:10.1016/j.proghi.2009.03.001. 19822255

[41] Koivusalo M, Welch C, Hayashi H, Scott CC, Kim M, Alexander T, Touret N, Hahn KM, Grinstein S 2010 Amiloride inhibits macropinocytosis by lowering submembranous pH and preventing Rac1 and Cdc42 signaling. J Cell Biol 188:547–563. doi:10.1083/jcb.200908086. 20156964 PMC2828922

[42] Diaz-Griffero F, Jackson AP, Brojatsch J 2005 Cellular uptake of avian leukosis virus subgroup B is mediated by clathrin. Virology 337:45–54. doi:10.1016/j.virol.2005.02.027. 15914219

[43] Miranda AF, Godman GC, Tanenbaum SW 1974 Action of cytochalasin D on cells of established lines. II. Cortex and microfilaments. J Cell Biol 62:406–423. doi:10.1083/jcb.62.2.406. 4214822 PMC2109385

[44] Coué M, Brenner SL, Spector I, Korn ED 1987 Inhibition of actin polymerization by latrunculin A. FEBS Lett 213:316–318. doi:10.1016/0014-5793(87)81513-2. 3556584

[45] Lim JP, Gleeson PA 2011 Macropinocytosis: an endocytic pathway for internalizing large gulps. Immunol Cell Biol 89:836–843. doi:10.1038/icb.2011.20. 21423264

[46] Mercer J, Helenius A 2008 Vaccinia virus uses macropinocytosis and apoptotic mimicry to enter host cells. Science 320:531–535. doi:10.1126/science.1155164. 18436786

[47] Shimojima M, Ströher U, Ebihara H, Feldmann H, Kawaoka Y 2012 Identification of cell surface molecules involved in dystroglycan-independent Lassa virus cell entry. J Virol 86:2067–2078. doi:10.1128/JVI.06451-11. 22156524 PMC3302412

[48] Moller-Tank S, Kondratowicz AS, Davey RA, Rennert PD, Maury W 2013 Role of the phosphatidylserine receptor TIM-1 in enveloped-virus entry. J Virol 87:8327–8341. doi:10.1128/JVI.01025-13. 23698310 PMC3719829

[49] Delpeut S, Sisson G, Black KM, Richardson CD 2017 Measles virus enters breast and colon cancer cell lines through a PVRL4-mediated macropinocytosis pathway. J Virol 91:e02191-16. doi:10.1128/JVI.02191-16. 28250131 PMC5411587

[50] Kondratowicz AS, Lennemann NJ, Sinn PL, Davey RA, Hunt CL, Moller-Tank S, Meyerholz DK, Rennert P, Mullins RF, Brindley M, Sandersfeld LM, Quinn K, Weller M, McCray PB Jr, Chiorini J, Maury W 2011 T-cell immunoglobulin and mucin domain 1 (TIM-1) is a receptor for Zaire Ebolavirus and Lake Victoria Marburgvirus. Proc Natl Acad Sci U S A 108:8426–8431. doi:10.1073/pnas.1019030108. 21536871 PMC3100998

[51] Kuroda M, Fujikura D, Nanbo A, Marzi A, Noyori O, Kajihara M, Maruyama J, Matsuno K, Miyamoto H, Yoshida R, Feldmann H, Takada A 2015 Interaction between TIM-1 and NPC1 is important for cellular entry of Ebola virus. J Virol 89:6481–6493. doi:10.1128/JVI.03156-14. 25855742 PMC4474285

[52] Evans JP, Liu SL 2020 Multifaceted roles of TIM-family proteins in virus-host interactions. Trends Microbiol 28:224–235. doi:10.1016/j.tim.2019.10.004. 31732320 PMC7018592

[53] Zheng K, Kitazato K, Wang Y 2014 Viruses exploit the function of epidermal growth factor receptor. Rev Med Virol 24:274–286. doi:10.1002/rmv.1796. 24888553

[54] Wang R, Wang X, Ni B, Huan CC, Wu JQ, Wen LB, Liao Y, Tong GZ, Ding C, Fan HJ, Mao X 2016 Syndecan-4, a PRRSV attachment factor, mediates PRRSV entry through its interaction with EGFR. Biochem Biophys Res Commun 475:230–237. doi:10.1016/j.bbrc.2016.05.084. 27208778

[55] Wang R, Wang X, Wu JQ, Ni B, Wen LB, Huang L, Liao Y, Tong GZ, Ding C, Mao X 2016 Efficient porcine reproductive and respiratory syndrome virus entry in MARC-145 cells requires EGFR-PI3K-AKT-LIMK1-COFILIN signaling pathway. Virus Res 225:23–32. doi:10.1016/j.virusres.2016.09.005. 27619841

[56] Araki N, Johnson MT, Swanson JA 1996 A role for phosphoinositide 3-kinase in the completion of macropinocytosis and phagocytosis by macrophages. J Cell Biol 135:1249–1260. doi:10.1083/jcb.135.5.1249. 8947549 PMC2121091

[57] Lindmo K, Stenmark H 2006 Regulation of membrane traffic by phosphoinositide 3-kinases. J Cell Sci 119:605–614. doi:10.1242/jcs.02855. 16467569

[58] Miyata Y, Nishida E, Koyasu S, Yahara I, Sakai H 1989 Protein kinase C-dependent and -independent pathways in the growth factor-induced cytoskeletal reorganization. J Biol Chem 264:15565–15568. 2670942

[59] Williamson CD, Donaldson JG 2019 Arf6, JIP3, and dynein shape and mediate macropinocytosis. Mol Biol Cell 30:1477–1489. doi:10.1091/mbc.E19-01-0022. 30969891 PMC6724687

[60] Zhao H, Guo X-K, Bi Y, Zhu Y, Feng W-H 2014 PKCδ is required for porcine reproductive and respiratory syndrome virus replication. Virology 468–470:96–103. doi:10.1016/j.virol.2014.07.040. 25155198

[61] Gao J, Xiao S, Xiao Y, Wang X, Zhang C, Zhao Q, Nan Y, Huang B, Liu H, Liu N, Lv J, Du T, Sun Y, Mu Y, Wang G, Syed SF, Zhang G, Hiscox JA, Goodfellow I, Zhou EM 2016 MYH9 is an essential factor for porcine reproductive and respiratory syndrome virus infection. Sci Rep 6:25120. doi:10.1038/srep25120. 27112594 PMC4845007

[62] Straight AF, Cheung A, Limouze J, Chen I, Westwood NJ, Sellers JR, Mitchison TJ 2003 Dissecting temporal and spatial control of cytokinesis with a myosin II Inhibitor. Science 299:1743–1747. doi:10.1126/science.1081412. 12637748

[63] Qiao S, Feng L, Bao D, Guo J, Wan B, Xiao Z, Yang S, Zhang G 2011 Porcine reproductive and respiratory syndrome virus and bacterial endotoxin act in synergy to amplify the inflammatory response of infected macrophages. Vet Microbiol 149:213–220. doi:10.1016/j.vetmic.2010.11.006. 21129861

[64] Wang X, Yang X, Zhou R, Zhou L, Ge X, Guo X, Yang H 2016 Genomic characterization and pathogenicity of a strain of type 1 porcine reproductive and respiratory syndrome virus. Virus Res 225:40–49. doi:10.1016/j.virusres.2016.09.006. 27619842

[65] Matanin BM, Huang Y, Meng XJ, Zhang C 2008 Purification of the major envelop protein GP5 of porcine reproductive and respiratory syndrome virus (PRRSV) from native virions. J Virol Methods 147:127–135. doi:10.1016/j.jviromet.2007.08.018. 17913250

[66] Ma H, Jiang L, Qiao S, Zhi Y, Chen XX, Yang Y, Huang X, Huang M, Li R, Zhang GP 2017 The crystal structure of the fifth scavenger receptor cysteine-rich domain of porcine CD163 reveals an important residue involved in porcine reproductive and respiratory syndrome virus infection. J Virol 91:e01897-16. doi:10.1128/JVI.01897-16. 27881657 PMC5244331

[67] Schmittgen TD, Livak KJ 2008 Analyzing real-time PCR data by the comparative *CT* method. Nat Protoc 3:1101–1108. doi:10.1038/nprot.2008.73. 18546601

[68] Reed LJ, Muench H 1938 A simple method of estimating fifty per cent endpoints12. Am J Epidemiol 27:493–497. doi:10.1093/oxfordjournals.aje.a118408.

[69] Hou J, Li R, Qiao S, Chen XX, Xing G, Zhang G 2020 Glycoprotein 5 is cleaved by cathepsin E during porcine reproductive and respiratory syndrome virus membrane fusion. J Virol 94:e00097-20. doi:10.1128/JVI.00097-20. 32102888 PMC7199402

[70] Jensen EC 2013 Quantitative analysis of histological staining and fluorescence using ImageJ. Anat Rec (Hoboken) 296:378–381. doi:10.1002/ar.22641. 23382140

[71] Wiesmann V, Franz D, Held C, Münzenmayer C, Palmisano R, Wittenberg T 2015 Review of free software tools for image analysis of fluorescence cell micrographs. J Microsc 257:39–53. doi:10.1111/jmi.12184. 25359577

[72] Wang X, Wei R, Li Q, Liu H, Huang B, Gao J, Mu Y, Wang C, Hsu WH, Hiscox JA, Zhou EM 2013 PK-15 cells transfected with porcine CD163 by PiggyBac transposon system are susceptible to porcine reproductive and respiratory syndrome virus. J Virol Methods 193:383–390. doi:10.1016/j.jviromet.2013.06.035. 23835031

